# Work restrictions among healthcare providers in a northern Italian public academic hospital: an observational study

**DOI:** 10.1186/s12913-025-12430-4

**Published:** 2025-02-20

**Authors:** Silvia Pazzaglia, Martina Bruno, Simone Gambazza, Filippo Binda, Alberto Bisesti, Jessica Graziella Calegari, Dario Laquintana

**Affiliations:** 1 Department of Healthcare Professions, Fondazione IRCCS Ca’ Granda Ospedale Maggiore Policlinico Milano, Via Francesco Sforza 35, 20122 Milan, Italy; 2 Department of Clinical Sciences and Community Health, Dipartimento Di Eccellenza 2023-2027, Laboratory of Medical Statistics, Biometry and Epidemiology “G. A. Maccacaro”, Università Degli Studi Di Milano, Via Celoria 22, 20133 Milan, Italy

**Keywords:** Healthcare providers, Work-fitness, Hospital personnel management, Occupational health, Public hospital, Italy

## Abstract

**Background:**

Reduced work capacity in public hospitals has organizational repercussions, given the aging population, the shortage of healthcare workers, and the greater demand for healthcare services. In this study, we analysed the characteristics of staff assessed as *"fit with restrictions"* at a public academic hospital in northern Italy. We also aimed to identify individual and work-related variables that may be associated with the probability and timing of being “*fit with restrictions”*.

**Methods:**

In this single-center observational study, sociodemographic data from staff employed in the Department of Healthcare Professions at our institution were analyzed using logistic regression to assess any associations between staff characteristics and the probability of being "*fit with restrictions"*. Additionally, a multivariable Cox proportional hazard model was fitted to investigate the potential association between staff characteristics and the timing of their first assessment as "*fit with restrictions"*.

**Results:**

The study population was 2251 employees of which 18.4% (415/2251) were *"fit with restrictions";* 56.1% (233/415) of nursing staff had at least one restriction, whereas 72.3% (300/415) of staff with restrictions had a permanent restriction. Sex was not associated with the probability of being "*fit with restrictions"* (odds ratio [OR] 0.75, 95%CI: 0.55 to 1.03). However, the probability was 22.9% lower (95%CI: 14.1% to 31.8%) for rehabilitation and technical healthcare staff compared to that of nurses and midwives. The Cox model showed an increase in the hazards of being "*fit with restrictions*" by a factor of 1.30 (95%CI: 1.02–1.68) for females.

**Conclusions:**

A significant proportion of nursing staff face mobility and posture restrictions, with older hires and greater seniority associated with higher probabilities of restrictions. These findings underscore the importance of addressing aging and workplace conditions in the public healthcare sector, particularly considering differences across job profiles and sex.

## Summary of findings

• Nursing staff (56.1%) face permanent mobility and posture restrictions, with handling and carrying being the most common restrictions; a notable 41.2% of staff have multiple restrictions.

• While sex is not significantly associated with higher odds of being "*fit with restrictions*", older hires and greater seniority leads to higher probabilities of having restrictions.

• Female workers have a 30% higher hazard of being "*fit with restrictions*", and the risk is greater in the first 15 years of employment, with significant differences observed between job profiles.

## Background

Work and health are deeply interconnected. Good health allows individuals to perform their job roles efficiently, contribute meaningfully to their communities, and find personal fulfilment. In turn, work provides a sense of purpose, financial stability, and social structure [1]. However, when health is compromised, the ability to work efficiently can decline, leading to reduced productivity, higher absenteeism, and long-term consequences for both employees and employers [2]. This connection brings to light the importance of assessing and maintaining fitness to work, which refers to an individual’s physical and mental capacity to meet the demands of their job [3, 4].

Reduced work capacity in public hospitals and other agencies has organizational repercussions, given the aging population in Italy [5], the shortage of healthcare workers [6], and the greater demand for healthcare services. In practice, in a hospital ward where staff members have work restrictions, those without restrictions must compensate for their colleagues, leading to an increased workload and higher stress levels. From the patients' perspective, a shortage of healthcare providers combined with the presence of staff with restrictions can result in inconsistent and unequal care, ultimately compromising both its quality and accessibility.

In 2022, the World Health Organization underscored the severity of the global healthcare workforce crisis. A key concern is the aging workforce, which threatens the long-term sustainability of healthcare services, as the growing difficulty in replacing retiring professionals risks compromising both efficiency and continuity of care [7]. The Organization for Economic Co-operation and Development reinforced this trend [8], estimating a shortage of approximately 1.2 million doctors, nurses, and midwives—driven by demographic shifts, including an aging population and the urgent need to replace a rapidly aging healthcare workforce.

In Italy, these demographic changes are characterized by increased life expectancy and an aging workforce [9], both of which have significant implications for the sustainability of the healthcare system [10, 11]. In 2019, the Italian population aged 65 and over, reached approximately 13.8 million, accounting for 22.8% of the total population—an increase from 12 million (20.3%) in 2009 [12]. However, this trend clashes with the ongoing shortage of healthcare providers, posing a critical challenge that could jeopardize both the well-being of workers and the long-term sustainability of the social and healthcare system [13]. The aging phenomenon is not limited to Italy but affects most European countries as well. Demographic projections indicate that Europe's workforce is also aging (e.g., Spain, Portugal, Greece, and Ireland), with a rising share of workers aged 55 and older [14].

Considering that older populations generally require more medical services due to age-related health issues, and that an increasing number of Italians under 65 are living with one or more chronic conditions [15], a significant portion of healthcare staff also faces chronic health issues or functional limitations, which may impact their ability to perform their duties effectively. Younger individuals are not exempt from these challenges, as they may require healthcare services less frequently but often have specific needs, such as preventive care and mental health support. Therefore, health assessment for fitness to work becomes an integral part of hospital staff and patient safety. Occupational health assessment in Italy is regulated by workplace safety legislation. Assessment is performed by an occupational physician at medical examination for job changes or upon worker request or other circumstances as defined by law [3]. A proactive focus on fitness to work ensures that employees are healthy enough to meet the demands of their roles while preventing work-related health issues, fostering a sustainable, balanced relationship between work performance and overall health.

Until 2016, the Italian healthcare sector experienced a steady decline, losing almost 21,508 healthcare staff, bringing the total number of employees—aged 50.6 years old on average—to just below 650,000. However, over the last four years, there has been a rapid increase of 32,332 employees, due to the new hiring policies introduced and measures to combat the pandemic [16]. Given that a significant portion of the workforce may be living with chronic health conditions, even among relatively young individuals [2], it is essential to prioritize and promote the well-being of both current and future healthcare providers. By identifying and addressing the challenges that healthcare staff face, the healthcare system can develop targeted strategies to support their mental and physical well-being. This, in turn, will enhance the overall quality of care they provide, ensuring a healthier workforce in the long term.

This study aims to investigate the characteristics of healthcare providers *"fit with restrictions"* employed in the Department of Healthcare Professions (DHP) in a public academic hospital. The objective is to identify personal factors (such as age and sex) and work-related variables (such as job type and seniority) that may be associated with the probability and time to fitness for work restriction.

## Materials and methods

### Study design

This single-center, observational study was carried out at the DHP, Fondazione IRCCS Ca' Granda Ospedale Maggiore Policlinico, Milan, with data collection completed in December 2023.

### Setting

Our Institution is a public academic hospital; its services cover a variety of specialties in interdisciplinary care, besides biomedical, clinical, and translation research. With more than 900 beds, an average of approximately 3 million outpatient services, around 46,000 admissions, and nearly 2 million diagnostic tests are performed each year. The hospital’s organizational model delivers personalized healthcare service, with a distributed responsibility according to the area of competence, and individual care planning for each patient. Within the Fondazione, the DHP is responsible for transversal processes and strategies, including healthcare, patient safety, and research into healthcare professions. The DHP also manages and coordinates nursing and midwife staff, healthcare technicians, rehabilitation, prevention, and assistive personnel. The Fondazione employs a total of 3,700 individuals, with 2,251 of those working specifically at the DHP.

### Data collection

The sociodemographic data of the DHP employees were extracted using an administrative electronic platform (Aliseo Human Capital Management, Windex Srl, Verona, Italy) to obtain department figures (as of 1 June 2023) for: sex, date of birth, date hired, professional qualification and type of work contract (permanent or temporary, full time or part time). These data are regularly updated to reflect any changes in an employee's career or status, ensuring completeness with no expected missing information. Data on fitness for work are recorded in hospital electronic records, along with assessment made by an occupational physician during follow-up visits, in accordance with Decree Law 81/2008 [3]. These records were retrieved for this study during June to December 2023.

The occupational health assessment for fitness to work is classified into four categories: "*fit", "fit with restrictions"*, *"temporarily unfit"* or *"permanently unfit".* The first category, "*fit"*, refers to employees who are fully capable of performing all the tasks required by their job without any modifications. The "*fit with restrictions"* category applies to workers who can carry out certain duties but may require specific modifications or accommodations to ensure their safety and well-being. For example, a staff member with a previous bone fracture may be temporarily restricted from performing tasks such as lifting heavy loads or assisting with patient mobility. These restrictions could be temporary or permanent, and the nature of the limitation is clearly defined in the assessment. The "*temporarily unfit"* category is for employees unable to fulfill their job duties for a limited time, with a reassessment scheduled at a later date to determine their ability to return to work. For example, a nurse with a severe hand dermatitis that prevents them from washing their hands or wearing gloves would be required to stay home until the condition is resolved. Lastly, the "*permanently unfit"* category refers to employees who are unlikely or unable to return to their previous role, often due to significant health concerns that make it impossible for them to resume their prior work responsibilities. For instance, a midwife who has lost their sight due to a degenerative disorder would be reassigned to alternative duties better suited to their abilities. The date of latest assessment for fitness for work restriction, the date of the first restriction, and the type of restriction (temporary or permanent) were retrieved for analysis, merging administrative data with employees’ records.

### Statistical analysis

Variables are expressed as mean and ± 1 standard deviation (SD) and as count and percentage (%); data are stratified by fitness for work. The two-sample Wilcoxon test was used to compare continuous variables and the Chi-square test statistics or Fisher exact test for categorical variables, as appropriate.

To investigate the potential association between staff characteristics and the probability of being *"fit with restriction",* a logistic regression model was fitted accounting for seniority, age at hiring, sex, and job profiles. Nineteen healthcare providers were grouped into 4 profiles according to current Italian healthcare staff profiling: [17] Nurses and Midwives (NM), Rehabilitation and Technical Healthcare Professions (RTHP), healthcare workers and staff with discontinued professions. The NM profile included nurses and midwives, the RTHP included health educators, physiotherapists, speech therapists, orthoptists, neuropsychomotor therapists, audiometrists, radiology technicians, neurophysiology technicians, medical lab technicians, and cardiovascular perfusionists. Discontinued professions denote assistants, general nurses, technicians, technologists and childcare nurses for which professional training courses are no longer conducted and the Italian healthcare system no longer hires [18–20]. Age at hiring and seniority were fitted using their non-linear functional forms as restricted cubic splines with four and three knots [21], respectively, based on the Akaike information criterion (AIC).

A multivariable Cox proportional hazard model was fitted to investigate the potential association between time to restriction and the staff characteristics entered into the logistic model, excluding seniority. Model’s assumptions were checked using smoothed scaled Schoenfeld residuals and formal tests, as proposed by Therneau [22]. Due to a mild violation of proportional hazard model assumption for age at hiring, the final model was stratified by time to restriction divided into two intervals: from the beginning to 15 years and after 15 years of service. Age at hiring age was fitted using three knots restricted cubic spline based on AIC. Each model was selected using the likelihood ratio test. Model validation and calibration with 500 bootstraps were performed reporting Somers’ Dxy and mean error, respectively. Model estimates are reported as odds ratio (OR) and hazard ratio (HR) with 95% confidence interval (CI). For all analyses, *P*-values were two-sided and *P* < 0.05 was considered statistically significant. All analyses were done using R Core Team [23] version 3.6.2, with *survminer* and *rms* packages added.

### Ethical statement

The local Ethics Committee, Milano Area B (Milano, Italy), has granted the study an exemption from the requirement for ethics approval and consent to participate. This exemption applies because the study involves observational research using pre-existing data on hospital staff, which does not necessitate ethical approval. The study was conducted in accordance with the principles of good clinical practice, the tenets of the Declaration of Helsinki, and European clinical practice.

## Results

In June 2023, a total of 2251 employees were working at the DHP staff and 18.4% (415/2251) were assessed "*fit with restrictions"* (Table 1). Their median with interquartile range follow-up time was 9.6 (2.4; 18.4) years.

**Table 1:** Healthcare staff characteristics

	***Fit with restrictions***	***Fit***	**Overall**	***P*-value**
N	415	1836	2251	
Age, yrs	53.9 (7.4)	41.4 (11.5)	43.7 (11.9)	< 0.001
Sex				0.139
Male	77 (18.6%)	401 (21.8%)	478 (21.2%)	
Female	338 (81.4%)	1435 (78.2%)	1773 (78.8%)	
Seniority, yrs	20.8 (9.3)	10.4 (10.1)	12.3 (10.7)	< 0.001
Age at hiring, yrs	33.2 (8.5)	31 (7.8)	31.4 (8)	< 0.001
Age at first work restriction, yrs	44.8 (8.2)	-	-	
*Healthcare Providers*				< 0.001
*Nurses and Midwives*				
Nurse	233 (20.1%)	924 (79.9%)	1157 (51.4%)	
Pediatric nurse	21 (12.4%)	148 (87.6%)	169 (7.5%)	
Midwife	24 (19.2%)	101 (80.8%)	125 (5.6%)	
*Rehabilitation and Technical Healthcare Professionals*				
Health Educator	-	3 (100%)	3 (0.1%)	
Physiotherapist	2 (4.8%)	40 (95.2%)	42 (1.9%)	
Speech Therapist	-	12 (100%)	12 (0.5%)	
Orthoptist	-	10 (100%)	10 (0.4%)	
Neuropsychomotor Therapist	-	7 (100%)	7 (0.3%)	
Audiometrist	-	6 (100%)	6 (0.3%)	
Radiology Technician	2 (2.8%)	70 (97.2%)	72 (3.2%)	
Neurophysiology Technician	2 (22.2%)	7 (77.8%)	9 (0.4%)	
Medical Lab Technician	8 (5.4%)	140 (94.6%)	148 (6.6%)	
Cardiovascular Perfusionist	-	3 (100%)	3 (0.1%)	
*Healthcare Workers*	65 (17.5%)	307 (82.5%)	372 (16.5%)	
*Discontinued Professionals*				
Assistant	33 (57.9%)	24 (42.1%)	57 (2.5%)	
General nurse	-	1 (100%)	1 (0%)	
Technician Assistant	4 (40%)	6 (60%)	10 (0.4%)	
Technician	-	2 (100%)	2 (0.1%)	
Childcare nurse	21 (45.7%)	25 (54.3%)	46 (2%)	
Work schedule				< 0.001
Full time	347 (83.6%)	1709 (93.1%)	2056 (91.3)	
Part time	68 (16.4%)	127 (6.9%)	195 (8.7)	

Data are presented as mean and ± 1 standard deviation (SD) and count and percentage (%). Row percentage for "*fit with restrictions*" and "*fit*" Healthcare providers are reported

Staff "*fit with restrictions"* was 12.5 years older (95%CI: 11.6 to 23.4) than *"fit"* staff. The age at hiring for those "*fit with restrictions"* was 2.1 years greater (95%CI: 1.2 to 3.0), and their seniority was 10.4 years greater (95%CI: 9.4 to 11.4) than *"fit"* staff. Males and females were equally distributed across the fitness for work categories (*P* = 0.139). In this sample, 600 out of 2,251 (26.7%) *"fit"* and 252 out of 415 (60.7%) *"fit with restrictions"* employees were aged 55 years or older.

**Fig. 1:**
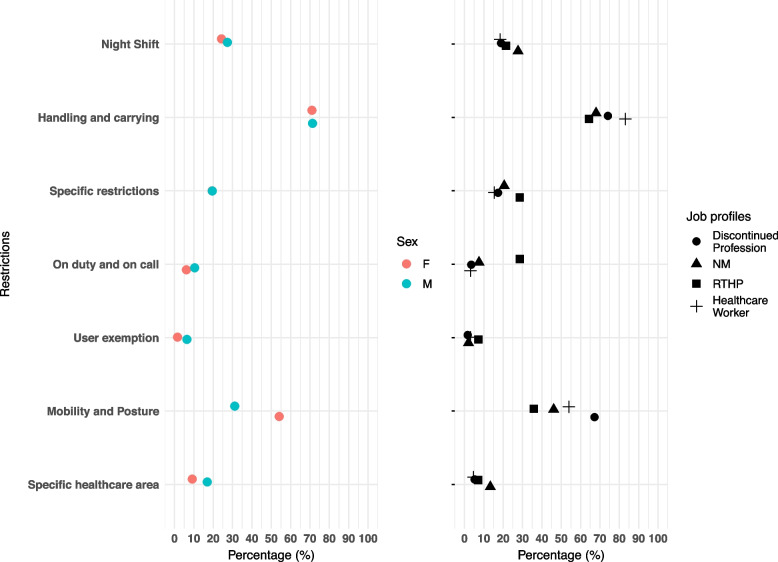
Left: Proportion of females (F, red dots) and males (M, blue dots) staff with specific work restrictions; Right: Proportion of staff with specific work restrictions

Over half (56.1%, 233/415) of the staff with at least one restriction were nurses, whereas 8% (33/415) were assistant personnel, and the majority of them was *"fit with restrictions"* (33/57, 57.9%). Overall, 72.3% (300/415) had a permanent restriction; those with a permanent mobility and posture restriction (161/300, 53.6%) outnumbered those with a temporary restriction, 46/115 (40%, *P* = 0.013). The most frequent restriction was handling and carrying, which equally affected females (71%, 240/338) and males (71%, 55/77, *P* = 0.94), as depicted in Fig. 1. A considerable number of staff (171/415, 41.2%) had at least two restrictions (Table 2). In the different job profiles, the percentage of staff with specific restrictions is almost identical, except for discontinued professions and RTHP, which show more restrictions compared to others under different mobility and posture and on duty and on call-restrictions, respectively 67% (39/58, *P* = 0.017) and 29% (4/14, *P* = 0.005) (Fig. 1).

**Table 2:** Fitness for work in staff with permanent and temporary restrictions

	**Permanent**	**Temporary**	**Total**	***P*-value**
N	300	115	415	
Restrictions				0.26
1	116 (38.7%)	52 (45.2%)	168 (40.5%)	
2	131 (43.7%)	40 (34.8%)	171 (41.2%)	
≥ 3	53 (17.7%)	23 (20.0%)	76 (18.3%)	
Mobility and Posture	161 (53.7%)	46 (40.0%)	207 (49.9%)	0.013
Handling and carrying	214 (71.3%)	81 (70.4%)	295 (71.1%)	0.86
Night Shift	72 (24.0%)	31 (27.0%)	103 (24.8%)	0.53
On duty and on call	19 (6.4%)	10 (8.7%)	29 (7%)	0.4
Specific healthcare area	27 (9.0%)	17 (14.8%)	44 (10.6%)	0.087
User exemption	6 (2.0%)	4 (3.5%)	10 (2.4%)	0.38
Specific restrictions	53 (18%)	28 (24%)	81 (19.5%)	0.12

Data are presented as count and percentage (%)

**Fig. 2:**
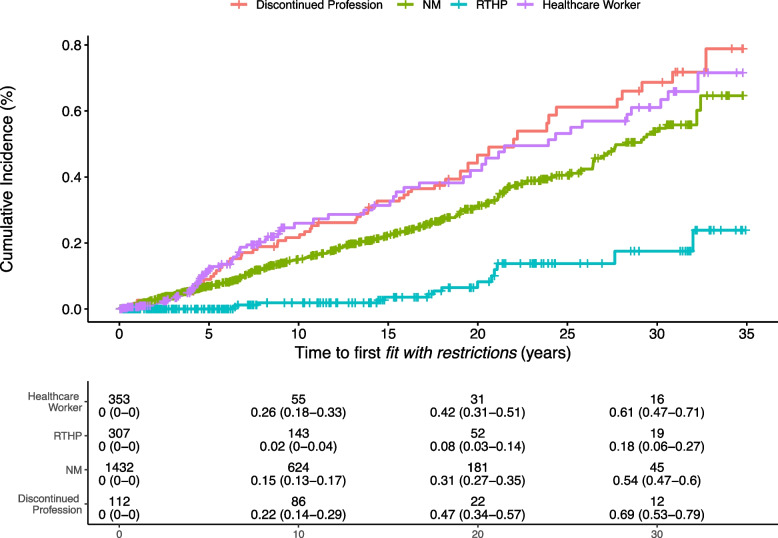
Cumulative Incidence Curves of staff* "fit with restrictions".* Numbers are people at risk at each considered time point with incidence estimates with 95% Confidence Intervals. One employee "*fit with restrictions"* after 39 years of service and one employee censored after 40 years of service were excluded from cumulative incidence presentation

At 10 years into their work life, 26% (95%CI:18 to 33 %) of healthcare workers were *"fit with restrictions".* Healthcare workers and those with a discontinued profession changed trajectory early, at about 5 years, compared to the NM profile (Fig. 2).

The logistic model showed that sex was not associated with the odds of being "*fit with restrictions"* (OR 0.75, 95%CI: 0.55 to 1.03; *p* = 0.074). At five years of employment, the odds increased by a factor of 5.39 (95%CI: 3.14 to 9.23; *p* < 0.001). Between five and ten years of employment, the odds increased by a factor of 3.47 (95%CI: 2.67 to 4.50; *p* < 0.001). At 10 years of employment, an additional 5 years is associated with a 1.76-fold (95%CI:1.43 to 2.17) increase in the odds of being "*fit with restrictions"* (*p* < 0.001). The model also showed that age at hiring was associated with the odds of being "*fit with restrictions"* (OR 2.31, 95%CI: 1.88 to 2.84; *p* < 0.001); for example, for woman hired in the NM profile at age 40 and 45 years with 10 years of experience, the probability is 29.1% (95%CI: 22.3 to 36.9%) and 37.3% (95%CI: 28.7 to 46.8%), respectively. No evidence of interaction was found between any of the covariates (*P* = 0.123). Regarding the probability of being "*fit with restrictions"* across job profiles, the model showed no difference between the discontinued professions and NM profiles (*P* = 0.581) or between NM and healthcare workers (*P* = 0.996). The probability was the same for staff with discontinued roles and healthcare workers (*P* = 0.829). However, the RTHP profile had a 22.9% lower probability of being "*fit with restrictions"* than the NM profile (95%CI: 14.1% to 31.8%; *p* < 0.001).

**Fig. 3:**
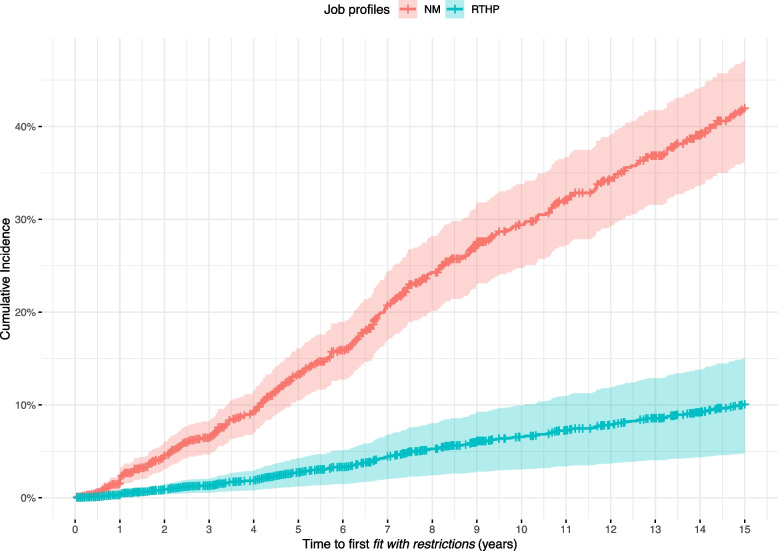
Cumulative Incidence Curves for Women in NM and RTHP profiles hired at Age 40 During the First 15 Years of Work as estimated from the Cox model. Bands are 95% Confidence Intervals

Among the characteristics entered into the Cox model, sex was associated with the hazard of being "*fit with restrictions"*: female sex increased the hazards by a factor of 1.30 (95%CI: 1.02 to 1.68; *p* = 0.038). The hazards ratio associated with the first assessment of "*fit with restrictions"* in the first 15 years of employment, assuming a hiring age of 40 years, was 3.88 (95%CI: 2.87 to 5.24), which was later mitigated (HR 1.43, 95%CI: 1.00 to 2.05). Figure 3 presents the cumulative incidence for being "*fit with restrictions"* at a given age for NM and RTHP profiles.

Compared with NM, discontinued professions had a 1.21-fold (95%CI: 0.91 to 1.61) increase in the hazard of being "*fit with restrictions"*, whereas healthcare workers had a 0.99-fold decrease (95%CI: 0.74 to 1.33). The only evidence of difference in the hazard of being "*fit with restrictions"* was between NM and RTHP (HR 0.19, 95%CI: 0.11 to 0.33; *p* < 0.001). The model showed no evidence of interaction (*P* = 0.1610).

Although validating data for prospective use was not necessary, the logistic model showed a corrected predictive discrimination of 0.67, with a slope shrinkage of 0.97 and an absolute calibration error of 0.005. The model will validate new data about 1.6% worse than on this dataset. The Cox proportional hazard model showed a corrected predictive discrimination of 0.42, with a slope shrinkage of 0.96; it will validate new data about 3.5% worse than on this dataset.

## Discussion

Our findings show that nearly one in five DHP staff members was *"fit with restrictions".* The most common restriction involved a reduced capacity for manual patient handling and carrying, followed by restrictions related to mobility and posture. The odds of being *"fit with restrictions"* were the same for men and women, although women were more likely than men to be assessed with restriction earlier. In addition, age at hiring was associated with time and the probability of being *"fit with restrictions"*. There was no evidence of difference in the odds of being *"fit with restrictions"* across job profiles, except that nurses and midwives were more likely to be *"fit with restrictions"* and to have it earlier than RTHP.

Occupational health assessment takes into account the changes in working capacity that occur due to physiological aging [24] accidents and illnesses [25, 26] or other personal factors [27], for example, over the normal course of work life. Workplace inclusion of employees with disabling injuries or illnesses remains a significant challenge in most industrialized countries, where musculoskeletal and mental health disorders are leading causes of work incapacity [28]. A 2015 study involving hospitals in Italy [29] and including medical staff reported that 11.8% (16,266/137,422) of employees were *"fit with restrictions"*, the most common of which regarded, as disclosed in our study, manual patient handling (49.5%) and posture (12.6%) [29].

Moving and lifting patients is one of the major risk factors for reduced work capacity among healthcare providers [30], and the number of workers exposed to such risk has increased considerably. A 2019 report published by the Italian National Institute for Insurance against Accidents at Work reported that musculoskeletal injury of the upper limbs and neck was the most common occupational injury in Europe, accounting for 45% of all occupational injuries [31]. Healthcare providers (e.g., nurses at most) are at high risk of being *"fit with restrictions"* due to musculoskeletal injury [31, 32], resulting from handling and moving non-autonomous patients, performing patient hygiene, and maintaining orthostatic posture for prolonged periods [33]. While we found no difference in the probability of being *"fit with restrictions"* for NM, healthcare workers, and the discontinued professions, the NM profile was more likely than the RTHP to be assessed as *"fit with restrictions"*. These findings may inform safe patient handling and mobility programs, research into ergonomic device design, and other technologies to create a safe and healthy workplace and facilitate the activities of staff with reduced functional ability. Rooms in high-risk departments that are fully equipped with ceiling hoists and aids for the proper mobilization of patients could be a strategy to ensure the availability and usability of such resources by healthcare providers. However, the literature is scarce on effective interventions to improve work environment, train older workers, provide ergonomic workplace conditions, and promote and maintain the health of the aging workforce [34]. We believe that there is a need to teach on university courses and training programs appropriate techniques for safe patient handling and mobility, precautions and procedures to minimize the risk of injury during patient moving and lifting [26, 30]. As suggested in the present study, offering this training during the initial years of employment could promote safe and appropriate practices, ultimately extending the period during which employees can work without restrictions.

The International Labor Organization has estimated that by 2025, there will be a 32% increase in the number of people aged over 55 years. Older people will make up approximately 30% of the population in Europe, where the current demographic trend is towards an increasingly aged workforce [35]. In the workplace sector, workers aged over 45 years are generally considered to be “older” [36]. In the coming years, the ongoing decline and aging of the population pose a significant threat to the long-term competitiveness of the European Union. The 2023 report on the impact of demographic changes in Europe highlights that member states are experiencing a significant reduction in the working-age population, which shrank by 3.5 million between 2015 and 2020. Projections indicate a further decline of 35 million people by 2050 [37]. In our study, the number of workers above 45 years is 50.5%, and it represents the 88.7% of staff *"fit with restrictions".* Overage employees may differ from their younger counterparts due to a number of physical/biological, psychological/mental, and social characteristics that influence their needs, expectations and challenges [38]. Physical functions, including sensory abilities, muscular function, aerobic capacity, reaction time and speed, immune response and the ability to maintain homeostasis all decline with age, and this deterioration becomes even more pronounced after the age of 50 [39]. In addition, overage workers usually have a higher prevalence of aging-related metabolic disorders such as abdominal obesity, hypertension, hyperglycemia, dyslipidemia, with reduced mobility and lower quality of life [40–42]. In contrast, the study by Nicholson et al. highlights that most individuals over 60, approaching the end of their careers, experience only a slight decline in cognitive abilities. This decline is often offset by well-established skills and the wealth of experience accumulated over the years. It is plausible that increased life expectancy, driven by improvements in quality of life, has slowed the natural age-related cognitive decline for a portion of this population [43].

The present findings support the importance of monitoring both subjective and objective work capacity in relation to worker age through targeted interventions. A previous study based on Italian data [29], reported that 24% of staff aged between 60 and 64 years had a reduced work capacity, whereas we found nearly twice (93/220, 42.3%) that proportion of the hospital staff aged 60–64 years in our sample. Bearing in mind that older workers may be more sensitive and less willing to accept change, it is highly probable that the majority will find it difficult to adjust to job modifications and new workplace conditions, as might be the case in our sample, aged 44 years on average. Occupational medicine interventions could help to promote and maintain functional ability, as well as identify the positive aspects of aging. A crucial aspect to implement is the promotion of continuous training opportunities that foster integration among different generations [44]. This reciprocal enrichment would allow older workers to share their accumulated expertise while benefiting from younger generations’ technological skills, such as computer proficiency, social media management, artificial intelligence applications, and digital tools. As workplaces evolve with the increasing integration of robotics, the demand for manual and repetitive tasks will decline. In contrast, there will be a growing need for specialized, cognitive, and digital skills. This shift underscores the importance of preparing the workforce for future challenges through strategic training and professional development initiatives [45].

National data for the Italian workforce show that being *"fit with restrictions"* differs by sex, with reduced work capacity and restriction more prevalent among women [29]. In detail, Italian Ministry of Health data (as of 31 December 2020) indicate that women hold 68% of jobs in the Italian national health system. The women’s share varies by healthcare service sector, however. For example, 77.7% of Italian nurses are women [46], which aligns with our finding (1030/1326, 77.7%). However, 81.4% (338/415) reported being *"fit with restrictions".* While we found no association between sex and the odds of being *"fit with restrictions"*, our data do show that the women were assessed *"fit with restrictions"* earlier than the men. A plausible explanation for this difference is a combination of biological, social, and workplace-related factors. Differences in aging may have a biological basis. For instance, women generally live longer than men [47], which does necessarily mean a high quality of life in older age: [48] compared with their male counterparts, women are more often affected by earlier onset of chronic illness (e.g., arthritis, arthrosis, osteoporosis) that leads to debilitating conditions [49]. In addition, women often have double the workload of professional and domestic work: 86.4% of women report family commitments, while 74.1% men do [50], indicating a considerable percentage of men sharing in domestic chores, nonetheless. The demands of domestic work may affect the working capacity of both men and women and depends on the work-life balance and an individual’s resources [51]. However, women, who make up a significant portion of the healthcare workforce, should receive greater attention from occupational medicine. This can be achieved not only by enhancing surveillance but also by encouraging their participation in physical activity programs, for example, to limit musculoskeletal disorders.

### Implications for practice

Protecting personnel against occupational risks and providing work modifications for employees *"fit with restrictions"* are the principles of workplace health promotion based on the joint effort of employers, workers, and society to improve worker health and well-being [52]. Our findings underscore the importance of implementing preventive measures and support across various job profiles in an effort to lower the risk of reduced work capacity and improve the overall well-being of healthcare providers. Joint meetings with occupational medicine, human resources, and hospital management on a regular basis have allowed us to monitor the staff and ensure that employees *"fit with restrictions"* are adequately reassigned within our hospital, thus balancing care needs with personal requirements. Monitoring and managing work restrictions through the development of digital databases—including key parameters such as company size, work area, job profile, gender, age, work-related and socioeconomic variables—could be instrumental for internal and inter-company analyses using standardized taxonomies. Additionally, it would facilitate data sharing at regional and national levels among occupational health services and healthcare institutions, promoting a coordinated, evidence-based approach.

It remains crucial to implement theoretical and practical training on proper posture and load-handling techniques to promote a safer and more sustainable work environment, especially during the first years of employment. For older staff, e.g., 60 years and above, assigning them to wards without night shifts would help minimize physical strain. Additionally, providing sufficient rest periods between shifts for all workers or strategic breaks to ensure adequate recovery time, reducing fatigue and minimizing the risk of injuries, would be essential for supporting both physical and mental recovery, ultimately enhancing overall work-life balance and well-being. It is essential to advance research on devices designed to reduce the workload associated with patient handling and to promote their integration into clinical practice. Generally, further research is essential to identify effective strategies for reassigning healthcare providers whose functional limitations affect their work, ensuring both workplace safety and efficiency. Additionally, future studies should consider the social, cultural, and organizational factors that contribute to functional decline, allowing for a more accurate assessment of the probability and timing of becoming *"fit with restrictions".*

The key question remains: are our findings unique, or do they reflect a nationwide trend that calls for urgent policy intervention by occupational medicine and surveillance? It would also be valuable to investigate conditions in residential care facilities, where healthcare providers operate in a distinct and demanding environment.

### Study strengths and limitations

Our data do not take into account previous employment or lifestyle factors or exposure to other risk factors present in the staff employed at public academic hospital. Therefore, the models may lack specificity, constituting a limitation of estimates, particularly as regards seniority. Staff working in a metropolitan area like Milan and in our hospital may face higher workloads and broader exposure to diverse conditions compared to those in rural or decentralized hospitals. As a result, our findings are most relevant to similar high-intensity healthcare settings. Nonetheless, our data provide valuable insight into work restrictions among staff in a large public academic hospital in Italy, addressing a gap in the literature and shedding light on this critical issue. Additionally, the multivariable logistic and Cox model demonstrate reliable estimates, thus offering stable measures of association for public health considerations.

## Conclusions

A considerable percentage of healthcare providers is *"fit with restrictions"*, a factor which could impact on human resource management within hospital wards. The risk of being *"fit with restrictions"* increases with age and seniority, and women tend to deal with restrictions earlier than men. Furthermore, nurses, midwives, and healthcare workers show a higher probability of being *"fit with restrictions".* These findings underscore the importance of addressing aging and workplace conditions in the public healthcare sector, particularly considering differences across job profiles and sex.

## Data Availability

The datasets used and analyzed during the current study are available from the corresponding author on reasonable request.
